# Effect of Soil Chemical Properties on the Occurrence and Distribution of Entomopathogenic Fungi in Portuguese Grapevine Fields

**DOI:** 10.3390/pathogens10020137

**Published:** 2021-01-30

**Authors:** Lav Sharma, Irene Oliveira, Fátima Gonçalves, Fernando Raimundo, Rupesh Kumar Singh, Laura Torres, Guilhermina Marques

**Affiliations:** 1 Centre for the Research and Technology of Agro-Environmental and Biological Sciences, University of Trás-os-Montes and Alto Douro (CITAB-UTAD), 5000-801 Vila Real, Portugal; ioliveir@utad.pt (I.O.); mariafg@utad.pt (F.G.); fraimund@utad.pt (F.R.); ltorres@utad.pt (L.T.); gmarques@utad.pt (G.M.); 2 Centre for Computational and Stochastic Mathematics, University of Lisbon (CEMAT-IST-UL), 1049-001 Lisbon, Portugal; 3 Centro de Química de Vila Real, Universidade de Trás-os-Montes e Alto Douro, Quinta de Prados, 5000-801 Vila Real, Portugal; rupesh@utad.pt

**Keywords:** *Beauveria*, biological control, entomopathogenic fungi, Hypocreales, *Metarhizium*, pest management, soil chemistry, vineyards

## Abstract

Entomopathogenic fungi (EPF) contribute to different ecosystem services. However, factors affecting their natural occurrences in soil remain poorly understood. In a previous study, 81 soil samples were subjected to insect baiting using *Galleria mellonella* and *Tenebrio molitor* to isolate EPF from Portuguese vine farms. Here, soils yielding any of the four common EPF, i.e., *Beauveria bassiana*, *Purpureocillium lilacinum*, *Metarhizium robertsii*, and *Clonostachys rosea* f. *rosea*, were correlated with their chemical properties. *Beauveria bassiana* was negatively affected by higher available P (*p* = 0.02), exchangeable K-ions (*p* = 0.016) and positively affected by higher soil pH_H_2_O (*p* = 0.021). High exchangeable K-ions inhibited *P. lilacinum* (*p* = 0.011) and promoted *C. rosea* f. *rosea* (*p* = 0.03). Moreover, high available K also suppressed *P. lilacinum* (*p* = 0.027). *Metarhizium robertsii* was inhibited by higher organic matter content (*p* = 0.009), higher C:N (*p* = 0.017), total N (*p* = 0.007), and exchangeable Mg-ions (*p* = 0.026), and promoted by higher exchangeable Na-ions (*p* = 0.003). Nonetheless, mean comparisons and principal component analysis suggested that higher soil pH and exchangeable Ca-ions have contrasting effects on EPF occurrences, as they promote *B. bassiana* and inhibit *M. robertsii*. Herbicides did not seem to affect EPF presence. Overall, this study is among the first reports on the effects of soil chemistry on EPF other than *Metarhizium*, and will facilitate biological pest management approaches.

## 1. Introduction

Entomopathogenic fungi (EPF) contribute to different ecosystem services. These include nutrient cycling, and regulatory roles, such as pest and plant disease control [1]. EPF can be considered as indicators of soil health [2] and, apart from assisting services for ecosystem functions, they contribute to the sustainable management of agroecosystems [3,4]. For example, EPF contribute to plant habitat adaptation against various biotic and abiotic stresses, and provide protection to plants against pathogens and pests [4]. EPF also interact with plants as growth promoters, beneficial rhizosphere colonizers, and biofertilizers [5]. These properties highlight their immense importance for environmentally friendly agriculture. Therefore, the factors that influence their diversity in cultivated soils are of paramount importance and should be thoroughly investigated.

Plants interact with their surrounding environment by releasing volatile organic compounds (VOCs) produced by leaves, flowers, and roots when exposed to abiotic (drought or mechanical damage) and biotic stress (due to insect herbivores, plant pathogens, or parasitic nematodes). VOCs influence the third trophic level organisms that feed on herbivores, i.e., entomopathogenic nematodes (EPN) and parasitoids/predators, both aboveground and belowground [6,7,8,9]. VOCs have been also tested in field conditions [10]. EPF, such as species from *Beauveria* Vuillemin (Hypocreales: Cordicipitaceae) and *Metarhizium* Sorokīn (Hypocreales: Clavicipitaceae) also release a range of VOCs [11,12,13,14,15], dictate parasitoids-mediated predation [12], and may repel weevils [14] and mollusks [15].

Both biotic factors (interactions with soil microbes and plants) and abiotic factors (temperature, soil texture, and agricultural inputs) influence the survival of EPF in soil [16]. Earlier research studies in this area have focused primarily on soil physicochemical properties. For example, a previous investigation considered the effects of soil pH, and texture (sand, silt, and clay contents) of Mediterranean soils on the occurrences of EPF [17]. Jabbour and Barbercheck thoroughly investigated effects of soil tillage intensity and cover crop on *Metarhizium* and other EPF during a three-year transition to organically managed farming in a feed grain rotation. They found an inverse relationship of *Metarhizium anisopliae* (Metschnikoff) with some soil chemistry traits and trace elements [18]. Clifton et al. measured EPF occurrence and abundance in conventional and organic fields in mid-western USA focusing on presence of *Metarhizium* and usage of herbicides and fungicides, and found a positive link with organic fertilizer and silt content [19].

Nonetheless, the knowledge of the effects of soil chemical properties on EPF diversity is very limited. Fewer soil chemical constituents were measured and, (a) either the objective was not to check their effect on the natural EPF diversity [20,21], or (b) studies primarily focused only on a few species, e.g., *M anisopliae* [18,19,22]. Those investigations brought a significant advancement in our knowledge on EPF ecology, however; understanding soil properties with respect to the occurrence of other EPF would enhance our knowledge about their survival and/or adaptability in a particular soil type. For example, soils with higher organic matter show higher biological activities, which in turn increase the abundance of EPF antagonists. On the contrary, soils poor in organic matter exhibit a reduced diversity and density of insects, i.e., potential hosts [23]. Studying different soil properties can therefore extend our knowledge about the soil ecology of EPF.

In this direction, only a few recent studies analyzed basic soil chemical constituents, such as total C and N, and correlated them with the occurrence of EPF other than *M. anisopliae* [23,24,25,26]. Few data are available on the natural presence of different EPF with respect to soil cation exchange capacity (a property which assists fungal conidia adsorption onto soils). In this study, we investigated the chemical properties of soils from Portuguese vine farms, by considering multiple variables such as: percentage of organic matter content (OM), total nitrogen (N), total organic carbon (C), available phosphorus (P), available potassium (K), exchangeable ions such as sodium (Na-ions), magnesium (Mg-ions), potassium (K-ions), and calcium (Ca-ions), pH, degree of base saturation (DBS), total acidity (TA), and effective cation exchange capacity (ECEC or CEC_e_). Their values were used to assess any impact on the occurrence of four widely known hypocrealean EPF: *Beauveria bassiana* (Balsamo), *Metarhizium robertsii* (Bischoff, Rehner and Humber), *Purpureocillium lilacinum* (Thom) Luangsa-ard, Houbraken, Hywel-Jones and Samson, and *Clonostachys rosea* f. *rosea* (Link) Schroers, Samuels, Seifert, and Gams, isolated previously [27]. Furthermore, to enhance variations in the soil chemistry, different soils were considered. The soils were (a) different in texture, i.e., coarse-texture or gross (high proportion of sand) or medium-texture (more balanced mixture of sand, silt, and clay); (b) sampled from varying habitat-types, i.e., cultivated vineyards or adjacent hedgerows which were mainly constituted of pine (*Pinus* spp. Linnaeus, Pinaceae) and oak (*Quercus* spp. Linnaeus, Fagaceae) trees; and (c) either treated with herbicides or left untreated.

## 2. Materials and Methods

### 2.1. Farms, Pests, and Sampling Site Description

The study was conducted in three Douro wine region farms of Portugal with *Vitis vinifera* L. varieties cultivation: Carvalhas (41°11′12.9″ N, 7°32′41.5″ W) (311.9 ha) (mix of red vine varieties: Sousão, Touriga Nacional, Tinto Cão), Granja (41°15′18″ N, 7°28′34″ W) (239 ha) (mix of white wine varieties: Moscatel, Fernão Pires, Verdelho), and S. Luiz (41°9′22″ N, 7°36′55″ W) (131.80 ha) (mix of red vine varieties: Touriga Nacional, Touriga Franca, Tinta Roriz, Viosinho, Tinta Barroca) during October and November 2012. The mean annual rainfall and temperature in the farms S. Luiz and Carvalhas ranged between 800 and 1000 mm and 14 and 16 °C. The farm Granja recorded 1000–1200 mm mean annual rainfall and temperatures ranging between 12 and 14 °C [28]. Farms are mainly managed through mating disruption for the control of the European grapevine moth *Lobesia botrana* (Denis and Schiffermüller) (Lepidoptera: Tortricidae). This is a key pest in these Portuguese vineyards and reduces up to 50% of the total crop yield at the time of harvest by rendering grape clusters susceptible to *Botrytis cinerea* Pers. (Helotiales: Sclerotiniaceae) resulting in primary and secondary rots [29]. These three farms were selected for their soil chemical properties as they exhibit relatively diverse landscapes (Shannon diversity-index for Carvalhas, S. Luiz, and Granja are 1.57, 1.09, 0.90; Eveness equitability index are 0.75, 0.53, and 0.46, respectively) [29], and the two baiting trap insects were used for soil EPF isolation in these three farms.

The herbicide applications in the farms were performed in March 2012, as follows: 2.5 L/ha GOAL^®^ SUPREME (48% p/v oxyfluorfen), and 4L/ha ROUNDAP^®^ SUPRA (37.7% p/p glyphosate) in S. Luiz; 3L/ha MARQUI^®^ (31% p/p glyphosate), 0.2 kg/ha KATANA^®^ (25% p/p flazasulfuron), and 3L/ha TOPZINA^®^ (45.7% p/p terbuthylazine) in Carvalhas; and 3L/ha FUEGO^®^ (22.3% p/v oxyfluorfen), and 3L/ha ROUNDAP^®^ SUPRA (37.7% p/p glyphosate) in Granja. No fertilizers were added in these farms for the mentioned year. More details about the usage of herbicide, the rapid texture of soil, and the chemical properties with respect to the sampling sites are provided in the Supplementary Table S1.

### 2.2. Soil Sampling

Soils were sampled from the above farms by digging up the top 20 cm of the soil surface using a soil core borer (width = 20 mm). Approx. 2 kg of soil was collected in total from each sampling site. For each sample, five subsamples were collected within an area of 0.25 m^2^ and mixed to obtain one sample per sampling site. Sampling sites were chosen at a distance of 20 m away. Additional details on the sampling scheme are provided in an earlier study [27]. Sampling tools were washed with 5% sodium hypochlorite (NaOCl) to avoid any possible fungal contamination between sites and all samples were treated independently. Approx. 1 kg of soil per sample was sent to the soil laboratory within the campus for soil analysis. Samples were air-dried and sieved with a 2 mm screen, and preserved for chemical analyses using conventional techniques. Remaining 1 kg portions of soil were processed immediately, i.e., within 24 h of the sampling, as described below.

### 2.3. Insect Rearing and Baiting

As a fungus can have different ecological roles, the possibility of a wrong functional annotation cannot be neglected [30]. A selective medium or a DNA-based approach is not sufficient to determine if a fungus is an entomopathogen or can just be a saprotroph or a phytopathogen. For example, a *Clonostachys* spp., *Clonostachys rhizophaga*, is a phytopathogen [31], whereas *C. rosea* f. *rosea* is an EPF [27]. Hence, insect baiting was preferred over soil suspension culture on selective media or a DNA based approach, as described [27,28]. Moreover, insect baiting is a widely accepted technique for the isolation of EPF from soil and many studies have demonstrated that it is better than soil suspension cultures on selective media [32,33]. However, using just one baiting trap insect can underestimate the presence of a particular EPF. For example, when the effectiveness of the baiting trap insects *Galleria mellonella* Linnaeus (Lepidoptera: Pyralidae) and *Tenebrio molitor* Linnaeus (Coleoptera: Tenebrionidae) for EPF isolation was compared, it was noticed that the former was more prone to infections by *B. bassiana*, and the latter to *M. robertsii* [27]. Therefore, the combination of both baiting trap insects was used in this study. Out of the 81 soil samples, 35 could yield at least one of the concerned EPF (*B. bassiana*, *M. robertsii*, *P. lilacinum*, and/or *C. rosea* f. *rosea*) that were studied for the soil chemical properties. Seven random soil samples that did not yield any EPF isolate were also analyzed, as negative controls. A total of 42 soil samples were examined, i.e., 21 from S. Luiz, 11 from Carvalhas, and 10 from Granja (see details in Supplementary Table S1). The insect larvae and their foods were bought from La Grilleria, Spain (www.lagrilleria.es) and were reared at 25 ± 2 °C, 50–60% RH, 16 h L:8 h D photoperiod.

For insect baiting, the soils were spread and left open overnight to equilibrate moisture content, adding the insect larvae the next morning for EPF isolation. The larvae of *G. mellonella* were given a heat shock in a water bath at 56 °C prior to baiting to reduce their tendency to form silk webs, which could hinder the fungal exposure during soil baiting, as suggested by Meyling and Eilenberg [34]. A total of 1 kg of soil was baited with 8 healthy late (fifth) instar larvae of each of the two baiting trap insects—*G. mellonella* and *T. molitor*. Therefore, 1 kg soil was divided into four 250 g bowls, each baited with 4 larvae of a same insect, accounting for 8 + 8, i.e., 16 larvae used in total per soil sampling site. Bowls were kept in an environmental camber (Panasonic MLR-352H-PE) at 22 °C and 85% relative humidity in dark. They were gently shaken periodically and kept upside down to ensure that the larvae reached most soil parts in the bowl [34,35]. The larvae were baited for three weeks as previously suggested [35], and were checked every second day for fungal growth. Larvae undergoing pupation, if any, were also tested for infection. The schedules were strictly monitored to ensure that larvae with foul smell or with nematodes emergence were constantly discarded.

### 2.4. Fungal Isolation and Screening

Insect cadavers were washed with 1% NaOCl for 3 min, followed by 3 distinct washes with sterilized water for 1 minute each. Oatmeal agar supplemented with 0.6 g/L cetyl trimethyl ammonium bromide (CTAB) (Sigma) and 0.5 g/L chloramphenicol (Acros) [36], and potato dextrose agar (PDA) supplemented with 0.05 g/L tetracycline (Acros) and 0.1 g/L streptomycin (Acros) were used for fungal isolation. Furthermore, for repeated culturing Oatmeal agar, PDA, and Sabouraud dextrose agar (SDA) (Prolabo) were used to obtain single colony cultures. To ensure the infectivity of the isolates, Koch’s postulates were verified as described in earlier studies [36,37,38]. In brief, sporulating fungi were excised from culture media and mixed in 0.02% Tween 20 solution (in sterilized distilled water). Fungal conidia concentration was adjusted to a 10^8^ conidia/mL and then 5 larvae of each insect were dipped into the solution for 3 s. The larvae were then incubated for a week at 22 °C in a petri dish at 85% RH, inside an environmental chamber, in the dark, and checked for mortality [27]. The tests for contamination were performed in parallel, as described and no evidence of external or cross-contamination was found [39]. Only those fungi that could kill the larvae within the first week were further considered as EPF.

### 2.5. Fungal Identification

The pure cultures of the EPF were then identified morphologically and through molecular methods. In brief, fungal morphology was first observed using a low magnifying stereomicroscope (Olympus SZX9, 40X), followed by their microscopic identification using a light microscope (Olympus BX51, 400X). For molecular identification, fungal DNA was extracted as mentioned by Möller et al. [40] and hard to crush mycelium was broken using beads, as described [41]. The nuclear internal transcribed spacer region of the fungal ribosomal DNA (*nrITS*) was amplified with PCR, sequenced, and aligned with existing type strain sequences available in Genbank using BLASTn, as described [27,42].

### 2.6. Soil Chemical Analyses

Different soil chemical properties were analyzed. In brief, soil pH was measured after preparing a soil–water suspension. Total organic carbon analyzer (Primacs SNC-100, Skalar Analytical, Breda, The Netherlands) was used to determine OM. Total N was estimated through Kjeldahl method and molecular absorption spectrophotometry was used for its quantification [43]. Extraction of P and K was performed using the Egnér–Riehm method, and for determination a spectrophotometer and a flame emission photometer (iCE™ 3300 AAS, Thermo Scientific^TM^, Breda, North Brabant, The Netherlands) were used, respectively. Atomic absorption spectrophotometry and subsequent ammonium acetate extraction at pH 7.0 were used to measure the amounts of exchangeable cations and bases [44]. Exchangeable acidity was measured using the titration method [45]. CEC_e_ was measured by summing exchangeable acidity and exchangeable bases, and the degree of base saturation was estimated by adding up the exchangeable bases, dividing it by CEC_e_, and then multiplying by 100.

### 2.7. Data Analyses

Normalities of the distributions analyzed and soil samples were grouped for the absence or presence of each EPF, independently. Student’s *t*-tests were used to determine differences between means of the soil variables for the presence/absence of each EPF. ANOVA was used to compare means between grouped factors. When normality was not assumed, nonparametric tests such as Kruskal–Wallis and Mann–Whitney were used instead, and subsequent unilateral significance (*p* < 5%) was obtained. In each nonparametric test for independent samples, the level of significance was based on the exact distribution of a statistical test, since the sample is small, sparse, and is poorly balanced. The Mann–Whitney *U* test with unequal sample sizes was not performed as the unequal group sizes may limit the statistical power. Instead, a Mann–Whitney *U* test with a Monte Carlo simulation (10,000 samples and a 0.95 confidence interval) was performed to evaluate the consistency of significant values from exact tests. Monte Carlo simulations were also used to gain better and more valid estimations of the obtained measures. Dimensionality reduction was performed on transformed data using Principal Component Analysis (PCA) and non-metric multidimensional scaling (NMDS). Software IBM SPSS Statistics version 22 (IBM, North Castle, Westchester, NY, USA) and XLSTAT version 2018.2 (Addinsoft, Bordeaux, Nouvelle-Aquitaine, France) were used to perform statistical data processing, and 3D scatter plots were made using NCSS version 12 (NCSS, Kaysville, Davis, UTAH, USA). Effect size analysis was performed using the software G*power ver. 3.1(University of Düsseldorf, Düsseldorf, Germany) [46].

## 3. Results

The OM and chemical properties of 42 soil samples were studied in terms of EPF occurrence. The corresponding statistics, *T*-values (*t*) or Mann–Whitney *U*-values (*U*) and *p*-values, are shown in Table 1. Details of the soil properties are provided in the Supplementary Table S1. All the significant values also resulted as significant by the Monte Carlo simulation.

### 3.1. Effects of Soils Chemical Properties, OM, and Herbicide Usage on EPF

**Figure 1:**
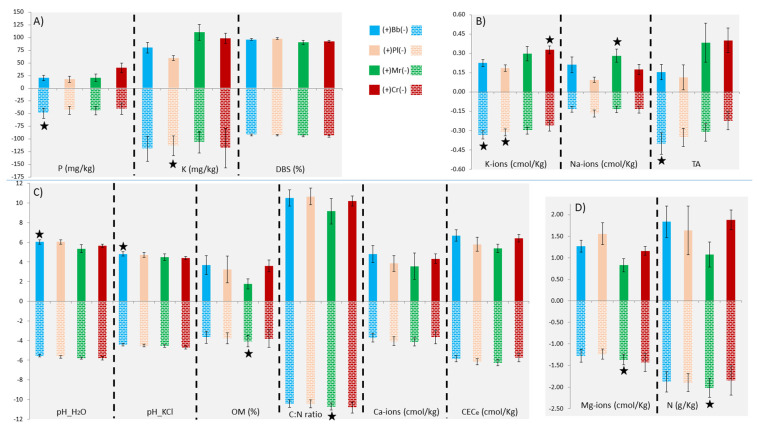
Effects of the soil properties on the occurrences on entomopathogenic fungi. Abbreviations B.b, P.l, M.r, and C.r stand for the entomopathogenic fungi *Beauveria bassiana*, *Purpureocillium lilacinum*, *Metarhizium robertsii*, and *Clonostachys rosea* f. *rosea*, respectively. Presence (+) and absence (-) are shown on the positive and negative Y axis, respectively. Unilateral significant observations, i.e., *p* < 5% is marked with an asterisk. Due to the difference in the scales used to access soil properties, soil variables are grouped according to the scales appropriate for their visualization as well as resolution. Overall, four different scales were used for the variables in the group (**A**–**D**). The units used for these scales, for e.g., cmol/kg, g/kg, mg/kg, and %, are mentioned alongside the measured soil variable. DBS, TA, OM, and CEC_e_ are: mean degree of base saturation, total acidity, organic matter, and effective cation exchange capacity, respectively.

*Beauveria bassiana* was associated with soils with less total acidity (*p* = 0.043) or higher pH_H_2_O (*p* = 0.021) (Figure 1, Table 1). Although some trends were noticed, in general, DBS and CEC_e_ did not affect EPF occurrences (Figure 1, Table 1). Among exchangeable ions, higher K-ions had a significant negative effect on the occurrence of *B. bassiana* (*p* = 0.016) and *P. lilacinum* (*p* = 0.011), and a significant positive effect on the occurrence of *C. rosea* f. *rosea* (*p* = 0.03). Excess of other exchangeable ions, i.e., Mg-ions and Na-ions, had inhibiting (*p* = 0.026) and promoting (*p* = 0.003) effects on *M. robertsii*, respectively. *Purpureocillium lilacinum* was also recovered from soils with higher Mg-ions, however, non-significantly (*p* = 0.077) (Figure 1, Table 1).

*Metarhizium robertsii* was inhibited by higher N (*p* = 0.007) and similarly, by higher C:N (*p* = 0.017). EPF in general were reported in soils with less N, P, and K, although there were a few marginal exceptions (Figure 1). Higher P inhibited *B. bassiana* (*p* = 0.02), and higher K reduced *P. lilacinum* (*p* = 0.027). Higher P had a minor inhibitory effect on *M. robertsii*, i.e., (*p* = 0.121) (Figure 1). *Metarhizium robertsii* was inhibited by OM (*p* = 0.009) whereas the other EPF did not seem to be affected by OM (Figure 1, Table 1). The mean values of all the soil variables with respect to the presence and absence of an EPF are shown in Table 1. Effect sizes for the significant observations are presented in Table S2. It was also noticed that the herbicide usage did not affect the presence of EPF as EPF could be isolated exactly equally from herbicide treated (*N* = 20/24) as well as untreated (*N* = 15/18) soils, i.e., 83.34% in each case (Table S1).

### 3.2. Biological Proximities and Related Factors

**Figure 2:**
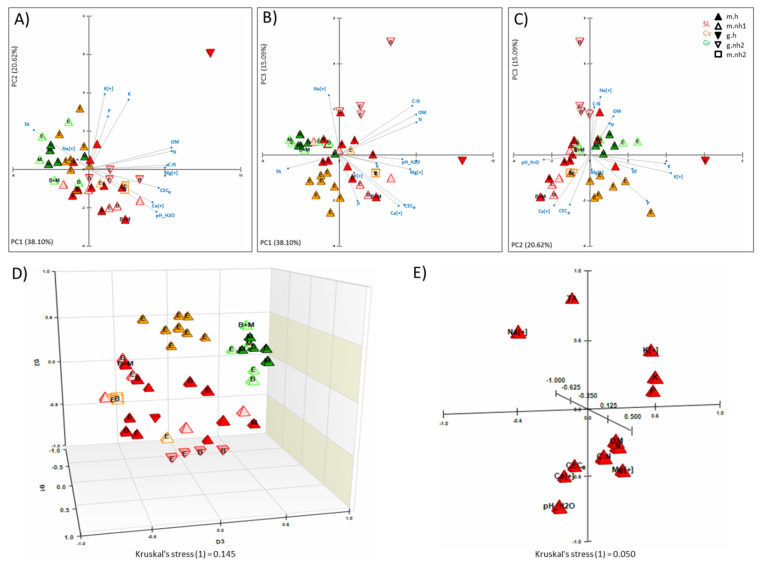
Clustering of soil samples harboring entomopathogenic fungi based on soil properties using principal component analysis (PCA) and non-metric multidimensional scaling (NMDS). (**A**) PC1 vs. PC2. (**B**) PC1 vs. PC3. (**C**) PC2 vs. PC3. (**D**) 3D-NMDS of soil samples. (**E**) 3D-NMDS of soil properties. Exchangeable cations are mentioned with a plus [+] sign. Farm types include S. Luiz (SL), Carvalhas (Cv), and Granja (Gr). Rapid texture is classified as gross (g) and medium (m). Entomopathogenic fungi are marked as E, if otherwise stated, i.e., *B. bassiana* (B), and *M. robertsii* (M). Other representations are soils with herbicide treatment (h), soils without any herbicide application and collected from vineyards (nh1), and soils from adjacent natural hedgerows which, by default, were never treated with herbicides (nh2).

Principal Component Analysis (PCA) was performed to understand the role of different soil properties on the occurrences of EPF and their subsequent clustering. As the significant effects of soil variables were only observed for *M. robertsii* and *B. bassiana*, the PCA was performed for three groups of EPF: *B. bassiana*, *M. robertsii*, and others (*P. lilacinum* and *C. rosea* f. *rosea*). First three components, i.e., PC1, PC2, and PC3 accounted for the 73.81% of the total variance, i.e., 38.10%, 20.62%, and 15.09%, respectively (Figure 2, Table S3). Principal component 1 could distinguish between the soils with and without *M. robertsii* (Figure 2A,B). It was noticed that *M. robertsii* isolations were negatively correlated with the higher amounts of OM, higher C:N, N, pH_H_2_O, Mg-ions, Ca-ions, and CEC_e_. As pH was negatively correlated with *M. robertsii*, it was shown that the soils higher in TA tended to favor *M. robertsii* isolations (Figure 2A,B). Principal component 2 clustered *B. bassiana* isolations with some exceptions (Figure 2A,C). It was found that *B. bassiana* was less prevalent in soils with higher K, P, TA, and K-ions. Soils with higher pH_H_2_O and Ca-ions instead tended to have higher occurrence of *B. bassiana* (Figure 2). Relationships among different soil variables are shown in Figure 2E. Factor loading and eigenvectors for different soil chemical characteristics are presented in the Supplementary Table S3.

## 4. Discussion

Soil characteristics can affect fungal communities and host–microbe interactions [20]. Studying soils where EPF spend a considerable part of their life cycle is of a high relevance for the management of their populations. In this study, soil physicochemical properties were analyzed for common soil EPF. *Beauveria bassiana* showed significant affinity to soils with less acidity or higher pH, a situation consistent with another study that also reported the association of *B. bassiana* with soils with higher pH [17,25]. In the present study, pH, TA, DBS, and CEC_e_ did not show a significant effect on other EPF. Previous investigations also reported no significant effects on important factors such as soil pH and CEC_e_ on *Metarhizium* occurrence [18,47].

Higher N tended to inhibit the occurrences of EPF, with the exception of *C. rosea* f. *rosea*, as higher N might have favored the growth of other fungi which could be competitors or consumers of EPF, or decomposers [48,49]. Our data showed that N, P, and K availability tended to reduce EPF occurrence, with some marginal exceptions (Figure 1). A negative correlation between *Metarhizium* and N content was noticed earlier [19]. It was also observed that fertilizers influence above- and belowground components which ultimately may reduce efficacy of biological control by EPF [50]. Koorem et al. reported that the abundance of soil arbuscular mycorrhizal fungi was negatively correlated with soil P [51]. Moreover, addition of NPK fertilizers was also reported to reduce the density of other entomopathogens such as nematodes [52]. Jaronski suggested that soils receiving high fertilizer input are dominated by bacteria [16]. Clifton et al. argued that soil microorganisms, particularly bacteria, exploit elevated nitrogen concentration and hence, outcompete EPF propagules for substrates [19]. In other studies, it was noticed that nutrient stressed environment, for e.g., soils with lesser N, may enhance EPF virulence and germination, as in the case of *Metarhizium* [24,53]. Moreover, EPF can mobilize insect-derived N in the scarcity of plant-derived N, and trade it for plant carbohydrates as endophytes [24,54]. Such property imparts an advantage for EPF over other soil microorganisms as lower soil nutrient levels reduce competition and antagonist presence [24].

Herbicides i.e., glyphosate, oxyfluorfen, flazasulfuron, and terbuthylazine applications, did not affect the field EPF occurrence. Various studies have reported negative effects of glyphosate [55] and oxyfluorfen [56] can significantly impact EPF vegetative growth and sporulation in-vitro. However, Clifton et al. also did not notice any significant impact of glyphosate and glufosinate-ammonium on EPF in field assays and also argued that herbicides do not affect the natural insect infection rates of EPF, such as *Metarhizium* [19].

Organic matter content had no effect on EPF occurrences apart from *Metarhizium* (Figure 1). An increase in OM subsequently increases CEC_e_, which enhances fungal conidia adsorption [17]. However, another study reported a negative association of OM with *Metarhizium*, comparative to ours [18]. Previous studies suggested that OM increases the biological activity in soils enhancing the growth of other saprotrophic fungi and eventually reducing the resources for EPF. *Beauveria* and *Metarhizium* are poor competitors for OM compared to saprotrophic fungi that are ubiquitous in soils [35,57]. Klingen et al. suggested that although a high OM may increase the EPF adsorption, these fungi maybe subsequently killed by saprotrophic fungi, in such soils [58]. Hence, it was not surprising that a higher C/N, resulting from a high OM, significantly increased soil fusaria in Portuguese vineyards [28]. Bidochka et al. also noticed that *Metarhizium* prefers agricultural and cultivated habitats that tend to have lower OM when compared with forest or semi-natural habitats [59]. We observed that *M. robertsii* could only be isolated from tilled vineyard soils and not from untilled hedgerows dominated by oaks and pine trees.

As the majority of soils had a medium texture (a balanced mix of sand, silt, and clay) rather than a gross texture (a high proportion of sand), we could not find a conclusive correlation for this parameter, although, it was noticed that *M. robertsii* was only isolated from medium-textured soils (Table S1). Garrido-Jurado et al. noticed that the availability of *Metarhizium* conidia was lower in sandy soils [20], and adequate sand content eventually promotes the conidia mobility and may promote percent infection [20,25]. Medium textured soils, with a balanced sand and clay content, can benefit EPF, as their adsorption onto clay particles can lead to a better nutrient availability for the fungus, due to enhanced iron solubility [20]. Clay, in fact, is used in many biological control formulations, particularly as coatings of clay/chitosan complex [60].

## 5. Conclusions

Two different approaches—comparisons of means and factor analyses—were adopted to study the variations in the chemistry of soils harboring different EPF. It was found that both approaches complement each other. Principal component analysis based on the soil chemical properties showed two factors which separated *M. robertsii* from *B. bassiana.* Properties such as higher soil pH and Ca-ions have contrasting effects as they both promote *B. bassiana* and inhibit *M. robertsii*. Moreover, different profiles were observed for each EPF. It was noticed that while soil OM, N, higher C:N, Mg-ions, and CEC_e_ inhibit *M. robertsii* the most, inhibition of *B. bassiana* mainly occurs in soils with higher acidity, K, P, and K-ions. Therefore, these chemical indicators can be used to predict the soil quality in terms of EPF, and subsequent soil amendments to be undertaken. However, a holistic approach is necessary to access the effects of such amendments on other beneficial soil organisms. Garrido-Jurado et al. emphasized the importance of investigating the retention and migration of the infective conidia in soil [20]. Apart from the soil physicochemical properties, electrostatic interactions, and substratum hydrophobicity can play important roles in fungal conidia retention and transport [20,28].

Predicting soil microbial quality based on soil chemical properties could be a promising approach in for sustainable agriculture, to develop methods such as integrated pest management. Both inundative and natural biological control through EPF has been studied in vineyards. Cozzi et al. reported significant mortality of *L. botrana* in Italian vineyards by *B. bassiana*, comparable to mortality by *Bacillus thuringiensis* Cohn (Berlinger) (Bacillales: Bacillaceae) [61]. Sharma et al. [41] investigated natural mycosis of vine mealybug *Planococcus ficus* (Signoret) (Hemiptera: Pseudococcidae) in vineyards by *P. lilacinum*. However, the success of biological pest control depends on a number of factors including the effects on non-target arthropods that must be taken into account during field inundative use of EPF. Some EPF, such as *Beauveria* and *Lecanicillium* W.Gams & Zare (Hypocreales: Cordycipitaceae) are also considered as plant disease (powdery mildew, rusts etc.) antagonists, acting through competition for resources, antibiosis, systemic resistance, or even parasitism against soil fungi [62,63,64]. Hence, such interaction with soil micro-organisms should be examined prior to EPF applications. Interestingly, EPF applications are compatible along with the use of mycoparasites, and in some cases they act in synergy against insect pests [65], probably, because fungal traits imparting entomopathogenicity are also involved in phytopathogen’s biocontrol [64]. Nonetheless, the EPF persistence should also be considered, as the ideal condition would be a longer persistence of a specialist EPF against a particular insect-pest, rather than a long-term persistence of a generalist EPF. According to Jaronski, one of the fundamental observations about the interactions of EPF with soil microbes is that non-sterile soils do not allow increase in EPF titers, exhibiting fungistasis [16]. However; recent studies have suggested long term persistence of *B. bassiana, M. anisopliae*, *B. brongniartii* in soils for over a year [27,65]. Apart from soil microbes, EPF are prone to mycophagy by soil nematodes and oribatid mites, protozoa, collembolans (springtails), enchytraeids, and earthworms. Luckily, evidences of EPF pathogenicity against earthworms and springtails are rare, if not absent [65]. To conclude, in plant protection practice, it is important to rely on a holistic approach to understand the relationships of organisms with the habitat, as well as among coexisting species. Further investigations on the ecology of the EPF and soil (micro)-biota would immensely benefit our understanding towards biological control of insect pests, as, for example, functional EPF diversity in vineyards (agricultural soils in this study) can be very different from that of oaks and pine trees (the major constituent of hedgerows in this study) [27,41]. Nonetheless, in parallel, such an approach can be extended to other beneficial soil microbes.

## Figures and Tables

**Table 1:** Statistical test, mean value for fungal absence and presence, and significance values for the effects of the different soil properties on the occurrences of entomopathogenic fungi.

Soil Properties	*Beauveria bassiana*				*Purpureocillium lilacinum*				*Metarhizium robertsii*				*Clonostachys rosea* f. *rosea*			
	MW-U ^a^	Mean for Absence	Mean for Presence	*p* ^a^	MW-U ^a^	Mean for Absence	Mean for Presence	*p* ^a^	MW-U ^a^	Mean for Absence	Mean for Presence	*p* ^a^	MW-U ^a^	Mean for Absence	Mean for Presence	*p* ^a^
P (mg/kg)	113	49.37 ± 52.57	20.38 ± 17.79	0.02	60	43.43 ± 48.61	17.95 ± 14.10	0.11	87	44.28 ± 49.48	20.99 ± 19.55	0.121	208	40.13 ± 47.55	40.6 ± 46.75	0.419
K (mg/kg)	140.5	119.59 ± 133.11	80.48 ± 37.29	0.097	43	113.90 ± 119.40	60 ± 11.22	0.027	78.5	106.81 ± 123.19	110.86 ± 0.77	0.07	182	118.98 ± 165.95	99.01 ± 48.01	0.193
OM content (%)	168	3.69 ± 3.28	3.7 ± 3.47	0.295	85	3.75 ± 3.37	3.24 ± 3.01	0.396	54.5	4.08 ± 3.45	1.77 ± 1.34	0.009	207	3.81 ± 3.79	3.6 ± 2.96	0.409
N (g/kg)	169	1.88 ± 1.26	1.83 ± 1.32	0.305	82	1.89 ± 1.28	1.64 ± 1.26	0.353	51.5	2.02 ± 1.29	1.08 ± 0.77	0.007	200.5	1.85 ± 1.44	1.88 ± 1.14	0.347
C:N	168	0.0104 ± 0.0017	0.01 ± 0.002	0.999	78	0.01 ± 0.001	0.01 ± 0.0016	0.298	60	0.01 ± 0.0018	0.01 ± 0.0011	0.017	165	0.01 ± 0.001	0.01 ± 0.001	0.097
pH_H_2_O	114.5	5.55 ± 0.67	6.04 ± 0.83	0.021	*t*^b^ = −1.06 df ^b^ = 40	5.66 ± 0.77	6.03 ± 0.5	0.147 ^b^	*t*^b^ = 1.336 df ^b^ = 40	5.77 ± 0.68	5.36 ± 1.03	0.094 ^b^	*t*^b^ = 0.596 df ^b^ = 40	5.78 ± 0.76	5.64 ± 0.75	0.277 ^b^
pH_KCl	*t*^b^ = −1.688 df ^b^ = 40	4.41 ± 0.71	4.82 ± 0.77	0.049 ^b^	*t*^b^ = −0.57 df ^b^ = 40	4.52 ± 0.77	4.72 ± 0.54	0.286 ^b^	110	4.55 ± 0.72	4.49 ± 0.91	0.346	*t*^b^ = 1.294 df ^b^ = 40	4.71 ± 0.68	4.41 ± 0.78	0.101 ^b^
TA	125	0.40 ± 0.45	0.15 ± 0.22	0.043	65.5	0.35 ± 0.42	0.12 ± 022	0.152	109	0.31 ± 0.41	0.38 ± 0.4	0.334	175	0.22 ± 0.29	0.4 ± 0.46	0.137
DBS (%)	133	91.90 ± 9.52	96.41 ± 5.76	0.068	65.5	92.67 ± 9.03	97.92 ± 3.96	0.152	103	93.87 ± 8.38	90.42 ± 10.47	0.265	192	94.49 ± 7.53	92.41 ± 9.57	0.261
Ca^2+^ (cmol/kg)	154.5	3.7 ± 2.06	4.81 ± 2.95	0.18	91	4.07 ± 2.47	3.85 ± 1.89	0.485	83	4.14 ± 2.18	3.57 ± 3.47	0.096	160	3.66 ± 2.33	4.33 ± 2.45	0.077
Mg^2+^ (cmol/kg)	162.5	1.28 ± 0.790	1.27 ± 0.49	0.242	55	1.24 ± 0.71	1.56 ± 0.57	0.077	65	1.36 ± 0.72	0.83 ± 0.40	0.026	201.5	1.43 ± 0.9	1.16 ± 0.5	0.356
K^+^ (cmol/kg)	110	0.33 ± 0.18	0.22 ± 0.10	0.016	35.5	0.31 ± 0.17	0.19 ± 0.06	0.011	115	0.30 ± 0.17	0.3 ± 0.14	0.408	142	0.26 ± 0.17	0.33 ± 0.15	0.03
Na^+^ (cmol/kg)	154	0.14 ± 0.12	0.21 ± 0.23	0.18	76	0.17 ± 0.17	0.09 ± 0.05	0.273	45	0.13 ± 0.15	0.28 ± 0.14	0.003	208	0.14 ± 0.11	0.18 ± 0.19	0.419
CEC (cmol/kg)	168	5.84 ± 2.41	6.69 ± 0.86	0.295	90	6.15 ± 2.75	5.80 ± 1.79	0.47	82	6.25 ± 2.48	5.36 ± 3.49	0.09	160	5.71 ± 2.91	6.4 ± 2.44	0.077

Abbreviations stand for: available phosphorous (P), available potassium (K), organic matter (OM), total nitrogen (N), carbon-nitrogen ration (C:N), total acidity (TA), degree of base saturation (DBS), exchangeable calcium ions (Ca^2+^), exchangeable magnesium ions (Mg^2+^), exchangeable potassium ions (K^+^), exchangeable sodium ions (Na^+^), and effective cation exchange capacity (CEC_e_). ^a^ Values are obtained by nonparametric statistical method, i.e., Mann–Whitney Test *U*-value (MW-U), unless stated otherwise. ^b^ Values are obtained by parametric statistical method, i.e., Student’s *t*-test, *T*-value (*t*), and degrees of freedom (df) are mentioned. Significant observations (*p* < 5%) are marked as bold.

## Data Availability

This study evaluates data generated in a previous study (https://doi.org/10.3897/mycokeys.38.26970) with new variables generated in this study. The soil chemistry data obtained in this study with respect to the microbes discussed were not published elsewhere. The Supplementary Data are deposited on the journal website for re-use and validation by research community.

## Supplementary Materials

The following are available online at https://www.mdpi.com/2076-0817/10/2/137/s1, Table S1: Chemical properties and other details of the soils sampled from the farms; Table S2: The effect sizes of significant observations with respect to the entomopathogenic fungi. Table S3: Details of principal component analysis.
